# High‐grade tumours promote growth of other less‐malignant tumours in the same prostate

**DOI:** 10.1002/path.5604

**Published:** 2021-01-26

**Authors:** Sofia Halin Bergström, Stina Rudolfsson, Marie Lundholm, Andreas Josefsson, Pernilla Wikström, Anders Bergh

**Affiliations:** ^1^ Department of Medical Biosciences, Pathology Umeå University Umeå Sweden; ^2^ Department of Surgical and Perioperative Sciences, Urology Umeå University Umeå Sweden

**Keywords:** high‐grade prostate cancer, low‐grade prostate cancer, angiogenesis, tumour‐associated macrophages, tumour instigator, Ki67, Factor VIII, CD68, experimental rat prostate tumour model, multifocal primary prostate cancer

## Abstract

Prostate cancer is a multifocal disease, but if and how individual prostate tumours influence each other is largely unknown. We therefore explored signs of direct or indirect tumour–tumour interactions in experimental models and patient samples. Low‐metastatic AT1 and high‐metastatic MatLyLu (MLL) Dunning rat prostate cancer cells were injected into separate lobes of the ventral prostate of immunocompetent rats. AT1 tumours growing in the same prostate as MLL tumours had increased tumour size and proliferation compared to AT1 tumours growing alone. In addition, the vasculature and macrophage density surrounding the AT1 tumours were increased by MLL tumour closeness. In patient prostatectomy samples, selected to contain an index tumour [tumour with the highest grade, International Society of Urological Pathology (ISUP) grade 1, 2, 3 or 4] and a low‐grade satellite tumour (ISUP grade 1), cell proliferation in low‐grade satellite tumours gradually increased with increasing histological grade of the index tumour. The density of blood vessels and CD68^+^ macrophages also increased around the low‐grade satellite tumour if a high‐grade index tumour was present. This suggests that high‐grade tumours, by changing the prostate microenvironment, may increase the aggressiveness of low‐grade lesions in the organ. Future studies are needed to explore the mechanisms behind tumour–tumour interactions and their clinical importance. © 2020 The Authors. *The Journal of Pathology* published by John Wiley & Sons, Ltd. on behalf of The Pathological Society of Great Britain and Ireland.

## Introduction

In most men with prostate cancer, multiple tumours are simultaneously present [1, 2]. If these tumours influence each other, and whether this is of any biological and/or clinical significance, is largely unknown. However, experimental evidence from our research group suggests that highly malignant tumours influence the rest of the prostate to form a tumour growth‐promoting environment [3, 4, 5]. As a consequence of this, one tumour could hypothetically influence the growth of another tumour in the prostate, through indirect effects in the prostate environment. The aim of this study was to explore this hypothesis.

Implantation of Dunning rat prostate tumour cells into the prostate of syngeneic and fully immunocompetent rats alters global gene expression profiles and tissue morphology in the non‐malignant prostate tissue. The nature and magnitude of these changes, named TINT changes (tumour instructed normal tissue), are related to tumour size, distance to the tumour, tumour growth rate, and metastatic capacity [3, 4, 5, 6, 7, 8, 9, 10]. For example, the growth of highly metastatic MLL tumours results in increased vascular density and accumulation of tumour‐promoting macrophages in the benign parts of the organ, and promotes a gene expression profile corresponding to immunosuppression [4, 8, 10]. In contrast, the growth of poorly metastatic AT1 tumours leads to a less pronounced increase in vascularity and macrophage infiltration in the adjacent *tinted* normal prostate tissue, and gives a gene expression profile instead suggesting immune activation [3, 4, 10]. Moreover, injection of a single dose of MLL tumour‐derived microvesicles into the normal prostate was able to precondition the prostate tissue and accelerate the growth of low‐malignant rat tumour cells implanted several days later [11].

In prostate cancer patients, the nature and magnitude of TINT changes in the benign parts of the prostate are related to patient outcome [4, 5, 12]. As individual prostate tumours influence the surrounding normal prostate tissue in ways that could promote or inhibit tumour growth and spread, interactions between tumours within the prostate are likely. Moreover, experimental studies of other tumour types have shown that aggressive tumours, by secreting factors systemically, can instigate the growth of indolent tumour cells at distant anatomical sites [13].

To explore prostate tumour–tumour interactions, we examined tumour characteristics in (1) rats with intraprostatic tumours of both AT1 and MLL compared with rats with AT1 or MLL tumours only, and (2) patients with multifocal prostate cancer of different histological grades.

## Materials and methods

### Animal experiments

AT1 and MLL rat prostate tumour cells were purchased from the European Collection of Cell Cultures (ECACC, Salisbury, UK; MLL # 94101454, AT1 # 94101449) and grown in RPMI 1640 + GlutaMAX (Gibco, Thermo Fisher Scientific, Waltham, MA, USA) supplemented with 10% foetal bovine serum (FBS) and 250 nm dexamethasone (Sigma Aldrich, St Louis, MO, USA) as described previously [10].

Immunocompetent and syngeneic adult Copenhagen rats (Charles River, Sulzfeld, Germany) were anaesthetised, and an incision was made in the lower abdomen to expose the ventral prostate lobes. To study animals with double tumours in the prostate, AT1 cells (4 × 10^4^ in 10 μl of RPMI) were injected into one of the ventral prostate lobes and MLL cells were injected into the contralateral lobe (2 × 10^3^ in 10 μl of RPMI) using a Hamilton syringe with a 30G needle. Control animals were injected with AT1 cells (4 × 10^4^ in 10 μl of RPMI) and vehicle (10 μl of RPMI) or MLL cells (2 × 10^3^ in 10 μl of RPMI) and vehicle (10 μl of RPMI). The animals were killed after 7 days (AT1 + MLL, *n* = 12; AT1 alone, *n* = 12; and MLL alone, *n* = 10) or 3 weeks (AT1 + MLL, *n* = 10; AT1 alone, *n* = 9; and MLL alone, *n* = 6) and the tumour‐containing prostates, the regional lymph nodes (iliac nodes), and lungs were removed and fixed in formalin and embedded in paraffin for further analyses.

In a separate experiment, AT1 cells (4 × 10^4^ in 10 μl) were injected into the ventral prostate (*n* = 12). Half of the animals were injected with MLL cells subcutaneously (2 × 10^5^ in 100 μl) and the other half served as controls and received a subcutaneous injection of vehicle (RPMI, 100 μl). The animals were killed after 7 days and the tumour‐containing prostates and the subcutaneous tumours were removed and formalin‐fixed.

Tissue sections were immunostained using a Ventana Benchmark ULTRA (Roche Diagnostics GMbH, Mannheim, Germany) with ULTRA CC1 (#950‐224; Roche) for antigen retrieval and primary antibodies for Ki67 (#ab16667, 1:100; Abcam, Cambridge, UK) or CD68 (#MCA341R, 1:200; AbD Serotec, Kidlington, UK). For Factor VIII immunostaining (#A0082, 1:1000; Dako Agilent, Santa Clara, CA, USA), sections were pretreated with ULTRA CC1 and Protease 1 (#760‐2018; Roche). All sections were visualised using the UltraView Universal DAB detection kit (#760‐500; Roche). The maximal tumour cross‐sectional area, the fraction of Ki67^+^ tumour cells, and the volume density (fraction) of CD68^+^ macrophages and Factor VIII^+^ blood vessels in the zone (0–0.5 mm) of normal prostate tissue surrounding the tumours were measured as described previously [4, 7, 8, 10].

All animal work was carried out in accordance with protocols approved by the Umeå Ethical Committee for Animal Research (permit number A 42‐15A).

### *In vitro* experiments

AT1 or MLL cells were incubated in serum‐free medium for 48 h and conditioned medium (CM) was collected, concentrated, and dialysed against PBS using a centrifugal filter with a 3 kDa cut‐off (Millipore, Burlington, MA, USA), giving secreted factors from the CM in PBS. Protein concentrations were determined using a DeNovix Spectrophotometer (DS‐11; DeNovix, Wilmington, DE, USA).

AT1 cells (10^4^ cells per well) were seeded in complete medium in a 96‐well plate and incubated at 37 °C overnight. Cells were carefully washed in PBS and incubated for 48 h with medium containing 1% FBS together with secreted factors from AT1‐CM, MLL‐CM (50 μg/ml total protein, 25% of the volume, respectively) or PBS as a control (25% of the volume).

In a co‐culture system, AT1 or MLL cells were plated in triplicates at 10^5^ cells per well of a 24‐well plate. AT1 cells (2.5 × 10^4^ cells per insert) were seeded in inserts (cell culture inserts, 0.4 μm pores; Corning/Falcon, Corning, NY, USA). After cell attachment, the inserts were placed above the AT1, MLL, or no cells as a control. All cells were grown in medium containing 5% FBS for 48 h.

The viability of AT1 cells, in the co‐culture inserts or stimulated with CM, was determined using the CellTiter‐Glo Assay (Promega, Madison, WI, USA) according to the manufacturer’s protocol. Luminescence was measured using the Spectramax i3X microplate reader (Molecular Devices, San Jose, CA, USA).

### Studies using patient samples

We selected samples with multifocal cancer from a cohort of patients receiving prostatectomy surgery at Umeå University Hospital during the period 2009–2019 (ethical approval number 03‐482 and 2013‐57‐31M; all patients included in the study gave their written consent). No treatment was given prior to prostatectomy surgery to any of the patients. All prostatectomy samples contained a satellite tumour with Gleason score (GS) 6 (3 + 3)/International Society of Urological Pathology (ISUP) grade 1 (*n* = 66) and an index tumour with either GS 8 (4 + 4)/ISUP grade 4 (*n* = 22), GS 7 (4 + 3)/ISUP grade 3 (*n* = 16), GS 7 (3 + 4)/ISUP grade 2 (*n* = 14) or GS 6 (3 + 3)/ISUP grade 1 (*n* = 14) situated elsewhere in the same prostate. The patients were staged as pT2–pT3 and had no signs of metastases. Pre‐operative serum PSA ranged from 2.3 to 20 μg/l.

Sections containing the index tumour and sections with the satellite were stained with antibodies against PSA (#A0562, 1:1000; Dako) and Ki67 [#790‐4286 (30‐9), pre‐diluted; Roche] as described previously [12]. The Ki67 labelling index was determined by counting at least 500 tumour cells situated in ten randomly selected areas within the tumour. The PSA staining index in the tumour was measured by multiplying staining intensity (graded from high = 3, moderate = 2, low = 1, and absent = 0) by distribution (1 = 0–25%, 2 = 26–50%, 3 = 51–75%, 4 > 75%), giving a score ranging from 12 (high intensity in most cells as in normal prostate glands) to 0 (no staining) [12]. In order to examine how different tumours affected their surroundings, the sections were also stained with antibodies against CD68 (#M0814, 1:2000, Dako; a pan macrophage marker) [14] and Factor VIII (#A0082, 1:1000, Dako; a marker for endothelial cells) [15], and the volume density (fraction of stained objects in the total tissue volume) of CD68^+^ macrophages and Factor VIII^+^ blood vessels was measured in tumour tissue and in a zone of histologically normal prostate tissue adjacent to the tumour (within 0–1 mm from the outer tumour border) using a square lattice mounted in the eyepiece of a microscope as described previously [4, 15, 16, 17].

### Statistical analysis

Values are represented as box plots and the Mann–Whitney *U* (MWU)‐test was used for comparisons between groups, or as mean ± SD with an unpaired *t*‐test for comparison between the groups. Mann–Whitney *U*‐tests and *t*‐tests gave very similar results (data not shown) and a *P* value less than 0.05 was considered significant. The Spearman rank correlation coefficient (*Rs*) was calculated for correlation studies. Statistical analysis was performed using Statistica 12.0 (StatSoft, Tulsa, OK, USA), SPSS Statistics 26 (SPSS Inc, Chicago, IL, USA), or GraphPad Prism 8 (GraphPad Software, San Diego, CA, USA).

## Results

### Intraprostatic MLL tumours increase the growth rate of intraprostatic AT1 tumours

First, we examined if two different rat prostate tumours growing simultaneously in the prostate could affect the growth rate of each other. Non‐metastatic AT1 tumour cells were injected into one lobe of the rat ventral prostate and metastatic MatLyLu (MLL) tumour cells were injected into the contralateral ventral prostate lobe (*n* = 12). Controls were injected with AT1 and vehicle (*n* = 12) or MLL and vehicle (*n* = 10) and animals were killed 7 days later.

**Figure 1:**
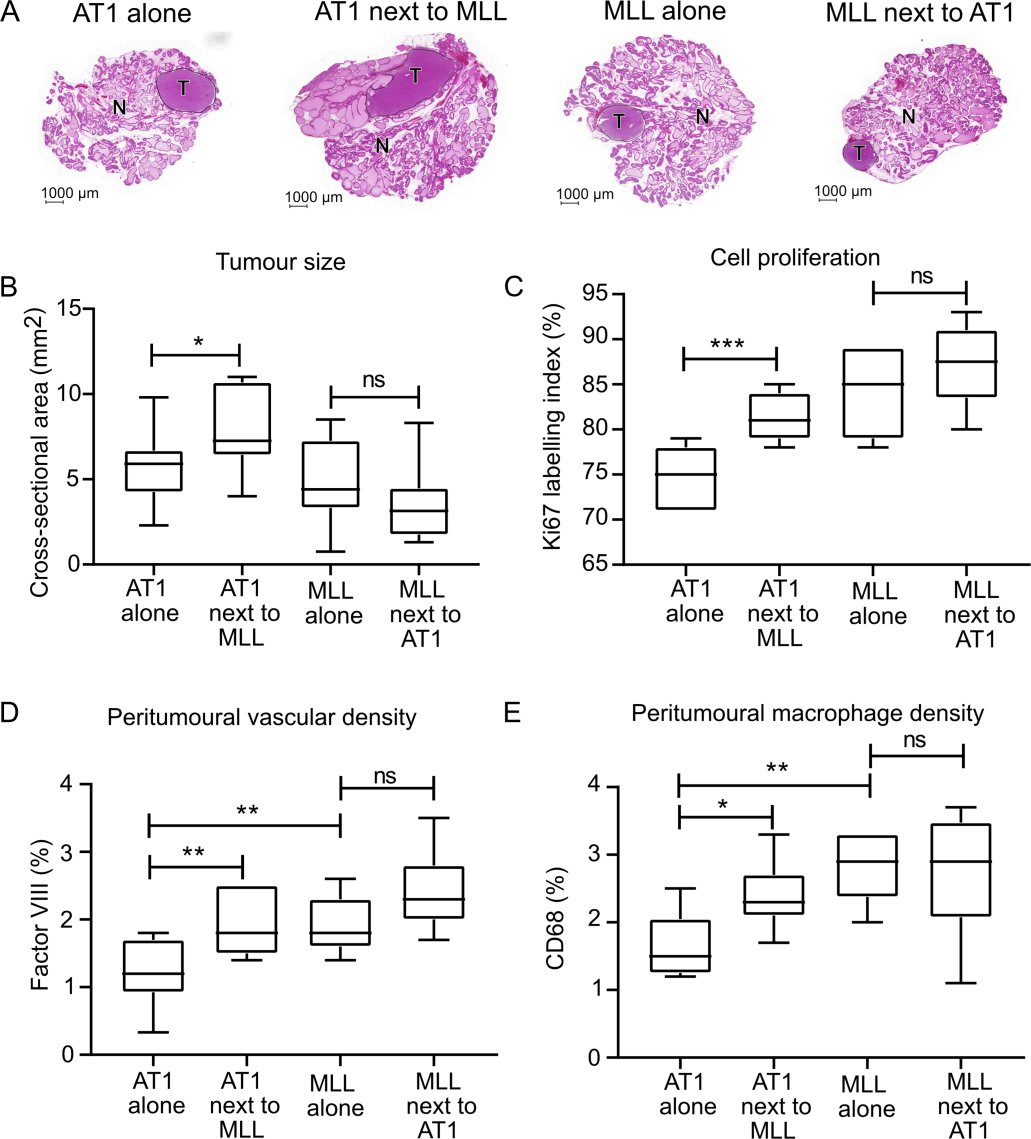
Analyses of intraprostatic MLL and AT1 tumours. (A) Haematoxylin and eosin staining of rat ventral prostate tissue with AT1 tumour alone, AT1 tumour when grown contralateral to MLL tumour, MLL tumour alone, and MLL tumour grown contralateral to AT1 tumour. Tumours (T) are encircled and surrounded by non‐malignant prostate tissue (N). (B–E) Graphs showing tumour size (B), proliferation (Ki67 labelling index) (C), peritumoural vascular density (Factor VIII, %) (D), and peritumoural macrophage density (CD68, %) (E) in AT1 tumours alone (*n* = 12), AT1 tumours next to MLL tumours (*n* = 12), MLL tumours alone (*n* = 10), and MLL tumours next to AT1 tumours (*n* = 12) illustrated with box plots. *p < 0.05, **p < 0.01, ***p < 0.001, ns = not significant, MWU test.

The MLL tumour size was unaffected by the presence of an AT1 tumour in the prostate (Figure 1). The AT1 tumour size, on the other hand, was significantly increased when grown next to the MLL tumour (Figure 1). In line with this, cell proliferation (Ki67 labelling index) was significantly increased in AT1 tumours growing in prostates where MLL tumours also were present (Figure 1 and supplementary material, Figure S1).

We then examined blood vessel (Factor VIII) and macrophage (CD68) densities in non‐malignant prostate tissue surrounding the tumours. As reported previously [4], single MLL tumours had significantly higher densities of blood vessels and macrophages in the benign parts of the prostate compared with single AT1 tumours (Figure 1 and supplementary material, Figure S1). However, when AT1 tumours were growing in the prostate with MLL tumours, the vascular and macrophage densities around the AT1 tumours (0–0.5 mm outside the tumour border) were increased and now similar to those of MLL tumours (Figure 1 and supplementary material, Figure S1).

### MLL tumours do not affect the metastatic capacity of AT1 tumours

In order to test if the increased growth rate of AT1 tumours was associated with increased metastases from AT1 tumours, we examined animals carrying tumours for 3 weeks.

In animals with single AT1 tumours, no metastases were found in the regional lymph nodes (0/9 animals), whereas all animals with single MLL tumours had detectable lymph node metastases (6/6 animals). In animals with both AT1 and MLL tumours, all animals (10/10 animals) had lymph node metastases, but they all had the histological phenotype of MLL (supplementary material, Figure S2). At this time point, no lung metastases were detected in animals with single AT1 tumours and were only occasionally found in animals with single MLL tumours or with both MLL and AT1 tumours. The few lung metastases found all had the MLL histological phenotype (data not shown).

The presence of an MLL tumour thus increased the growth rate of AT1 tumours but probably not their capacity to metastasise to lymph nodes and lungs, at least not as far as could be observed during the current time period.

### Intraprostatic MLL tumours stimulate AT1 tumours through local effects

**Figure 2:**
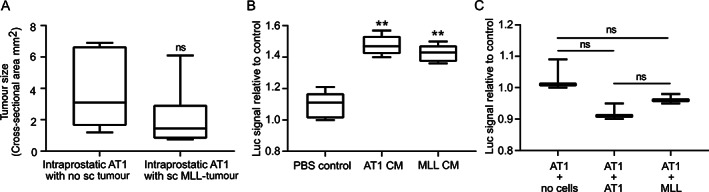
Systemic and direct effects of MLL tumour cells on AT1 tumour cell growth. (A) Tumour size of intraprostatic AT1 tumours grown with or without subcutaneous MLL tumours, (*n* = 6 per group) illustrated with box plots. ns = not significant, MWU test. (B) Cell viability *in vitro* of AT1 tumour cells grown for 48 h in medium with 1% FBS stimulated with secreted factors (in PBS) from AT1 conditioned medium (CM), or MLL CM relative to PBS stimulated controls using the CellTiter Glo assay (*n* = 5 in each group). **p < 0.01, MWU test. (C) Viability of AT1 tumour cells co‐cultured *in vitro* for 48 h in medium with 5% FBS together with AT1 cells (AT1 + AT1), or MLL cells (AT1 + MLL) relative to no cells (AT1 + no cells) using the CellTiter Glo assay (*n* = 3 in each group; ns = not significant, MWU test). The Transwell co‐culture assay allowed paracrine signalling between AT1 cells in the upper inserts and cells/medium in the lower chambers (0.4 μm pore size).

We tested if the growth stimulatory effect of MLL on AT1 tumours was locally or systemically provided. In this experiment, MLL tumours growing subcutaneously reached an average cross‐sectional area of 14 mm^2^, which is larger than those examined in the prostate. However, subcutaneous MLL tumours did not stimulate AT1 tumour growth in the prostate compared with controls (Figure 2). This suggests that the growth stimulatory effect of intraprostatic MLL on intraprostatic AT1 is likely a local rather than a systemic effect.

To explore if MLL tumours could stimulate the growth of AT1 tumours directly, we tested if conditioned medium (CM) from MLL cells affected AT1 viability *in vitro*. MLL‐CM increased AT1 cell viability compared with controls, but this effect was similar to the effect mediated by AT1‐CM (Figure 2). Moreover, in a Transwell co‐culture system, paracrine signals from MLL or AT1 cells did not affect AT1 viability compared with controls (Figure 2), suggesting that direct tumour–tumour cell interactions were minor.

### Low‐grade tumours in prostate cancer patients have higher proliferation when a high‐grade tumour is present in the prostate

We also examined signs of potential tumour–tumour interactions in selected prostatectomy samples in patients with two tumours (*n* = 66). All patients had an ISUP grade 1 satellite tumour and were divided into four groups based on the ISUP grade of the index tumour (ISUP grade 1–4) also present in the prostate.

In the index tumour, tumour cell proliferation (Ki67 labelling) significantly increased with increasing ISUP grade (Table 1 and supplementary material, Figure S3). Interestingly, proliferation was also significantly increased in ISUP 1 satellite tumours in patients with index ISUP grade 3 or 4 tumours compared with patients with index ISUP grade 1 or 2 tumours (Table 1 and supplementary material, Figure S3). In line with the animal experiments, this suggests that high‐grade tumours can stimulate the proliferation of adjacent low‐grade tumours also in patients.

**Table 1:** Cell proliferation and PSA staining score in patients with index tumours of different ISUP grades.

	Index tumour ISUP grade			
	1 (*n =* 14)	2 (*n =* 14)	3 (*n =* 16)	4 (*n =* 22)
Cell proliferation index tumour (Ki67 %)	2.1 ± 1.1	6.4 ± 2.7^*^	9.1 ± 3.3^*,†^	17 ± 10^*,†,‡^
Cell proliferation ISUP grade 1 satellite tumour (Ki67 %)	2.2 ± 1.6	2.9 ± 1.4	4.3 ± 1.6^*,†^	5.0 ± 1.0^*,†^
PSA staining score index tumour (0–12)	11 ± 1.7	8.3 ± 1.9^*^	7.0 ± 1.3^*,†^	6.8 ± 1.3^*,†^
PSA staining score ISUP grade 1 satellite tumour (0–12)	11 ± 1.5	11 ± 1.5	11 ± 1.5	11 ± 1.2

Values are mean ± SD; significantly different (*p* < 0.05, *t*‐test) than cases with *index tumour ISUP grade 1, ^†^index tumour ISUP grade 2, or ^‡^index tumour ISUP grade 3.

ISUP, International Society of Urological Pathology.

Hypothetically, high Ki67 labelling in low‐grade tumours could therefore indicate the presence of a high‐grade tumour elsewhere in the prostate. To examine this further, we screened available diagnostic needle biopsies from our ISUP 3 and 4 index cases for individuals where the diagnostic biopsies only showed ISUP grade 1 cancer. Ki67 labelling was significantly higher in ISUP 1 diagnostic biopsies from patients identified with grade 3 or 4 tumours after prostatectomy than in cases that remained as grade 1 also in the prostatectomy samples (6.0 ± 2.5, *n* = 7 versus 3.6 ± 1.5, *n* = 9, *p* < 0.05, respectively; mean ± SD, *t*‐test). There was no significant difference in the duration between primary diagnosis and prostatectomy in the grade 3 or 4 cases compared with the grade 1 cases (8.9 ± 3.9 versus 8.1 ± 7.0 months, respectively; mean ± SD, *p* = 0.82, *t*‐test). This suggests that high proliferation in low‐grade tumours could be a marker of a concurrent high‐grade tumour in the prostate.

As shown previously [12], the PSA staining score in the index tumour decreased significantly with increasing ISUP grade (Table 1 and supplementary material, Figure S3), showing that dedifferentiated high‐grade tumours have higher proliferation and lower PSA expression (*Rs* = −0.639, *p* < 0.0001). The most pronounced decrease in tumour cell PSA staining was seen between ISUP 1 and ISUP 2; that is, when some tumour glands have dedifferentiated and developed Gleason grade 4 patterns. However, the PSA staining score in the ISUP grade 1 satellite tumour was not affected by differences in the ISUP grade of the index tumour (Table 1 and supplementary material, Figure S3). As long as the tumour glands were categorised as Gleason grade 3 (ISUP 1), they maintained high PSA staining.

### Low‐grade tumours in prostate cancer patients have higher vascular and macrophage densities when a high‐grade tumour is present in the prostate

Having found that cell proliferation in low‐grade satellite tumours was related to the aggressiveness of the index tumour, we examined whether this was also associated with changes in vascular and macrophage densities as seen in the animal model. For this purpose, we only examined cases with index tumours of ISUP grade 1 or ISUP grade 4.

**Figure 3:**
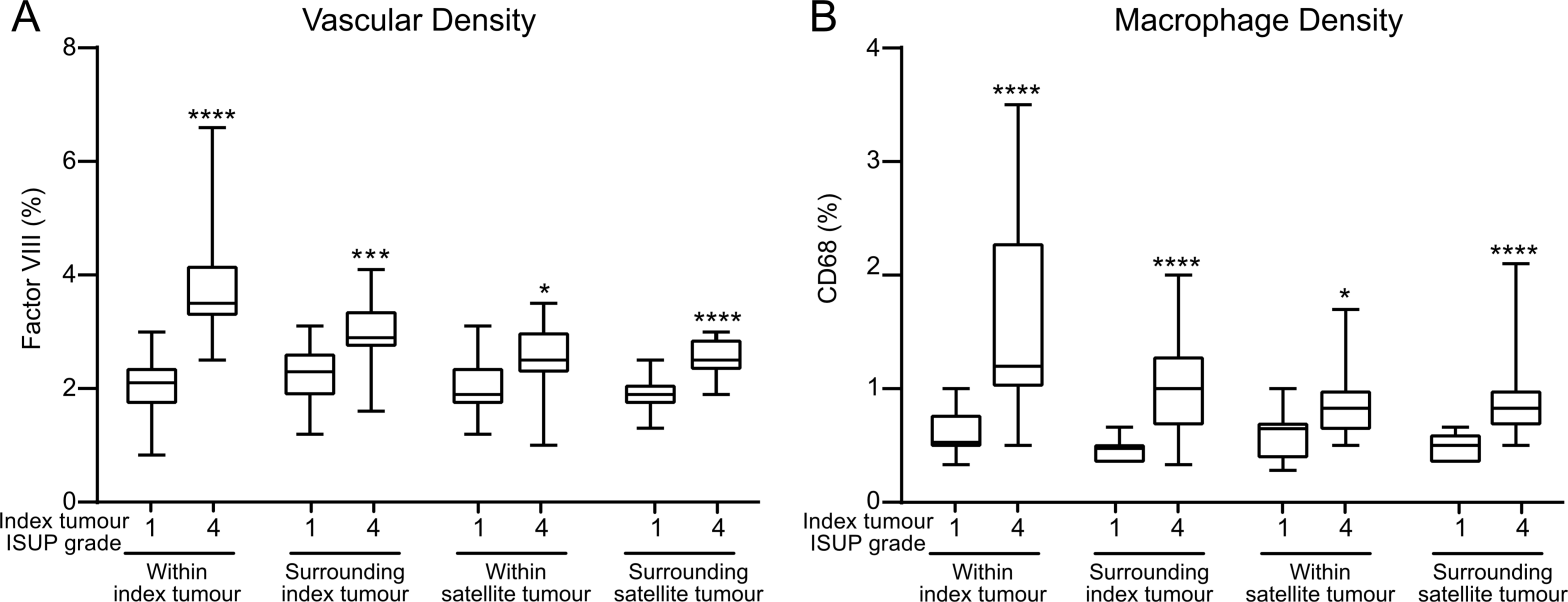
Vascular and macrophage densities in prostatectomy patient samples. (A) Vascular density (Factor VIII, %) and (B) macrophage density (CD68, %) within index tumours, surrounding index tumours, within satellite tumours, and surrounding satellite tumours in cases with ISUP grade 1 index tumours (*n* = 14) compared with cases with ISUP grade 4 index tumours (*n* = 22). *p < 0.05, ***p < 0.001, ****p < 0.0001, MWU test.

The density of Factor VIII‐stained blood vessels was higher within and around ISUP grade 4 index tumours than in ISUP grade 1 index tumours (Figure 3 and supplementary material, Figure S3), suggesting that high‐grade tumours secrete factors stimulating vascular growth. Similarly, the vascular density within and surrounding ISUP grade 1 satellite tumours was significantly increased in patients with ISUP grade 4 index tumours compared with patients with ISUP grade 1 index tumours (Figure 3 and supplementary material, Figure S3). Furthermore, Ki67 labelling in satellite tumours correlated with the vascular density in the normal tissue surrounding the tumour (*Rs* = 0.536, *p* = 0.001), but not with the vascular density inside the tumour (*Rs* = 0.238, *p* = 0.15). Taken together, this suggests that a high‐grade tumour stimulates vascular growth not only within and outside itself but also within and outside a low‐grade tumour elsewhere in the prostate.

Our previous studies have suggested that tumour‐induced vascular growth could be mediated by macrophages [4, 7]. The density of CD68^+^ macrophages was higher within and surrounding ISUP grade 4 index tumours than in ISUP grade 1 index tumours (Figure 3 and supplementary material, Figure S3). Correspondingly, the macrophage density within and surrounding ISUP grade 1 satellite tumours was significantly increased in patients with ISUP grade 4 index tumours compared with patients with ISUP grade 1 index tumours (Figure 3 and supplementary material, Figure S3).

Changes in proliferation, vascularity or macrophage density in the satellite tumours were not related to the size of the ISUP 4 index tumour (cross‐sectional area ranging from 0.2 to 1.7 cm^2^; data not shown) or to the distance between the index and satellite tumour (ranging from 0.5 to 2.5 cm; data not shown). This suggests that satellite tumours were more affected by index tumour grade than by index tumour size or distance between the tumours.

## Discussion

Here we have shown that an aggressive and metastatic rat prostate tumour increased the growth rate, but not obviously the metastatic capacity, of a less aggressive tumour in the same prostate. Consistent with the findings in experimental models, high‐grade cancers seem to stimulate growth of low‐grade tumours in patients, as indicated by the step‐wise induction of cell proliferation in ISUP grade 1 satellite tumours in relation to increasing ISUP grade and proliferation rate of the nearby index tumour.

The mechanisms explaining how more aggressive tumours accelerate the growth of less aggressive tumours are unknown. Tumour cell‐to‐cell interactions could be involved, but do not appear to be the most obvious explanation based on the result in the current study, as AT1 cell growth *in vitro* was unaffected when co‐cultured with MLL or AT1 cells in a Transwell assay, and AT1‐CM was equally effective as MLL‐CM in stimulating AT1 cell growth. Other possible mechanisms that involve indirect effects through non‐malignant cell types instead seem more plausible. Here we show increased macrophage and vascular densities in the non‐malignant tissue surrounding aggressive tumours, both in the experimental models and in the patients, which probably form a growth‐stimulating environment reaching also nearby less aggressive tumours.

The capacity of AT1 tumours to metastasise to the lymph nodes and lungs was not affected by the presence of MLL tumours, at least not at the time point studied. Further studies are needed to examine if metastatic tumours can affect the invasive and metastatic properties of less metastatic tumours within the same prostate.

In animal experiments, various highly aggressive tumour types, called instigators, have been shown to secrete factors into the circulation that are able to increase the growth of indolent tumours at distant anatomical sites [13, 18]. The instigator‐promoted growth was shown to be indirectly mediated by attracting bone marrow‐derived myeloid cells and platelets to the tumour site, and thereby stimulating angiogenesis [13, 18, 19]. Such tumour instigation may also occur in patients [20].

Factors systemically secreted by an aggressive prostate tumour could also possibly affect other tumours in the prostate. However, as subcutaneous MLL tumours did not stimulate AT1 tumour growth in the same manner as MLL tumours grown in the prostate, it appears that MLL‐induced changes in the local prostate microenvironment are more important for intraprostatic AT1 tumour growth than systemic instigation. This is in line with previous results showing that MLL tumours induce tumour‐promoting TINT changes in the benign parts of the prostate, for example increased blood supply, increased lymphangiogenesis, and an M2‐dominated inflammation, that facilitate subsequent growth and spread of the MLL tumour [3, 4, 7, 8, 10]. In addition, tumour‐derived MLL microvesicles also induce tumour‐promoting changes in the non‐malignant prostate tissue that facilitate the growth of less malignant tumours [11]. Aggressive tumours likely induce multiple changes in the non‐malignant surrounding tissue and in remote organs, such as the lymph nodes [8, 10]. The role of such changes in tumour progression and/or as prognostic markers needs to be further examined.

In line with the findings in experimental models, low‐grade satellite tumours in patients had increased macrophage and vascular densities, both inside and surrounding the tumours, if growing in the presence of a high‐grade index tumour. Presumably, a high‐grade index tumour promotes tumour growth by facilitating blood supply, through increased macrophage infiltration and vascular growth, in the non‐malignant prostate tissue surrounding both the index tumour itself and the satellite tumour. These observations could be of high biological and clinical importance.

Most patients with prostate cancer have a multifocal and polyclonal disease [1, 21, 22, 23]. Prostate cancer is diagnosed by taking tiny needle biopsies from the prostate. Such biopsies sample less than 1/1000 of the prostate volume. As the disease is often multifocal and as imaging cannot always guide biopsies towards the most malignant part of the most advanced tumour present [24], the most aggressive lesions may sometimes be undetected. Our study suggests that relatively high Ki67 labelling in an ISUP grade 1 tumour, and/or increased vascular and macrophage density around it, could indicate the presence of an undetected high‐grade (ISUP grade 3 or 4) tumour area/clone elsewhere in the prostate. This hypothesis now needs to be validated in a larger cohort of patients.

Currently, the prognosis in prostate cancer is to a large extent evaluated by characterising the so‐called index tumour (the tumour with the highest histological grade and the largest size). The clinical importance of additional low‐grade satellite tumours is assumed to be minimal [1]. The present study, however, suggests that all ISUP grade 1 satellite tumours are not equal. Their cell proliferation rate may differ at least two‐fold. Whether this is of any importance is unknown. In general, index ISUP grade 1 tumours seldom form metastases; are slow‐growing; and are considered good candidates for surveillance [1, 25]. However, in a cohort of prostate cancer patients diagnosed with needle biopsies and managed by surveillance, 27% of the ISUP grade 1 patients had a Ki67 labelling index greater than 5%, a cell proliferation rate associated with an increased risk of disease progression [26]. Similarly, in a cohort of ISUP grade 1 cases, diagnosed at trans‐urethral resection of the prostate and managed by watchful waiting, an increased risk of progression was seen if Ki67 was above 2.7% [12, 16]. In the same cohort, a high (above median) density of Factor VIII‐stained blood vessels in the benign parts of the prostate was associated with an increased risk of disease progression [16]. It is, however, unknown if these observations indicate that some ISUP grade 1 tumours are aggressive, or if they indicate the presence of an undetected high‐grade tumour (ISUP grade > 2) elsewhere in the prostate.

The current findings raise the question of whether an ISUP grade 1 tumour could, under the influence of other more aggressive cancers (in the prostate or in other organs), progress into a potentially lethal cancer. Epidemiological evidence suggests that some ISUP 1 tumours progress to higher grades [27], but the mechanisms involved are unknown. Further studies to identify tumour‐derived factors that stimulate a growth‐promoting microenvironment are needed [2, 8, 10]. Are growth‐promoting factors exclusively derived from high‐grade tumours or can they be produced by other pathological processes? In the current study, the effects of high‐grade lesions on other less malignant tumours may have been underestimated, as we cannot exclude that one effect of increased cell proliferation could be dedifferentiation and an increase in ISUP grade in satellites. Interestingly, in a patient where the origin of the lethal prostate metastases was traced back to the prostate, containing multiple tumours, the systemic metastases came from an ISUP 1 tumour area, whereas the index ISUP 4 tumour only formed regional lymph‐node metastases [28]. Hypothetically, this could be an example of clinically significant instigation and/or the TINT effect in a prostate cancer patient.

In summary, the present study suggests that the biological and clinical significance of tumour–tumour interactions, causing an altered local environment in patients with multifocal prostate cancer, could be of larger importance than recognised previously.

## Author contributions statement

SHB, SR and ML conceived and carried out animal and *in vitro* experiments. AJ collected patient data. SHB, PW and AB conceived, analysed, and interpreted animal and patient data. All the authors were involved in writing and revising the manuscript and gave approval of the submitted manuscript.

## Supporting information


**Supplementary figure legends**
**Figure S1.** Immunostaining of intraprostatic rat tumours
**Figure S2.** Morphology of lymph node metastases in rats
**Figure S3.** Immunostaining of prostatectomy patient samples
