# Supplementary material for: High‐grade tumours promote growth of other less‐malignant tumours in the same prostate
Source: J Pathol. 2021 Jan 26;253(4):396–403. doi: 10.1002/path.5604 (PMC7986692; doi:10.1002/path.5604)
Supplement: Supplementary file 1 — Supplementary figure legends Figure S1. Immunostaining of intraprostatic rat tumours Figure S2. Morphology of lymph node metastases in rats Figure S3. Immunostaining of prostatectomy patient samples [file PATH-253-396-s001.zip › supp5604-sup-0001-FiguresS1-S3/path5604-sup-SuppFigLegs.docx]

**High-grade tumours promote growth of other less-malignant tumours in the same prostate**

S Halin Bergström *et al. J Pathol* DOI: 10.1002/path.5604

**Supplementary figure legends**

**Figure S1**. **Immunostaining of intraprostatic rat tumours.** Sections from AT1 tumours growing alone, AT1 tumours growing next to MLL tumours, MLL tumours growing alone, and MLL tumour growing next to AT1 stained for Ki67, Factor VIII, and CD68. AT1 tumours growing adjacent to MLL showed increased Ki67 staining within the tumours and increased Factor VIII and CD68 staining at the tumour border compared to AT1 tumours growing alone.

**Figure S2.** **Morphology of lymph node metastases in rats.** Haematoxylin-eosin staining of sections from regional lymph nodes in animals carrying (A) a single AT1 tumour in the prostate, (B) a single MLL tumour in the prostate, and (C) both AT1 and MLL tumours in the prostate (low and high magnification). (D) Morphology of MLL and AT1 growing next to each other in the prostate (low and high magnifications). The animals with MLL tumours formed lymph node metastases with a MLL morphological phenotype but metastases with an AT1 morphology were not observed.

**Figure S3.** **Immunostaining of prostatectomy patient samples.** Sections from patients with ISUP 1 or ISUP 4 index tumours with ISUP1 satellite tumours. Sections are stained for Ki67, PSA, Factor VIII and CD68. The presence of an index ISUP4 tumour appear to increase Ki67 labelling of tumour cells, and Factor VIII or CD68 positive cells at the tumour border in its ISUP1 satellite, compared to that in satellites adjacent to ISUP1 index tumours.
