# Supplementary figures and images for: High‐grade tumours promote growth of other less‐malignant tumours in the same prostate
Source: J Pathol. 2021 Jan 26;253(4):396–403. doi: 10.1002/path.5604 (PMC7986692; doi:10.1002/path.5604)

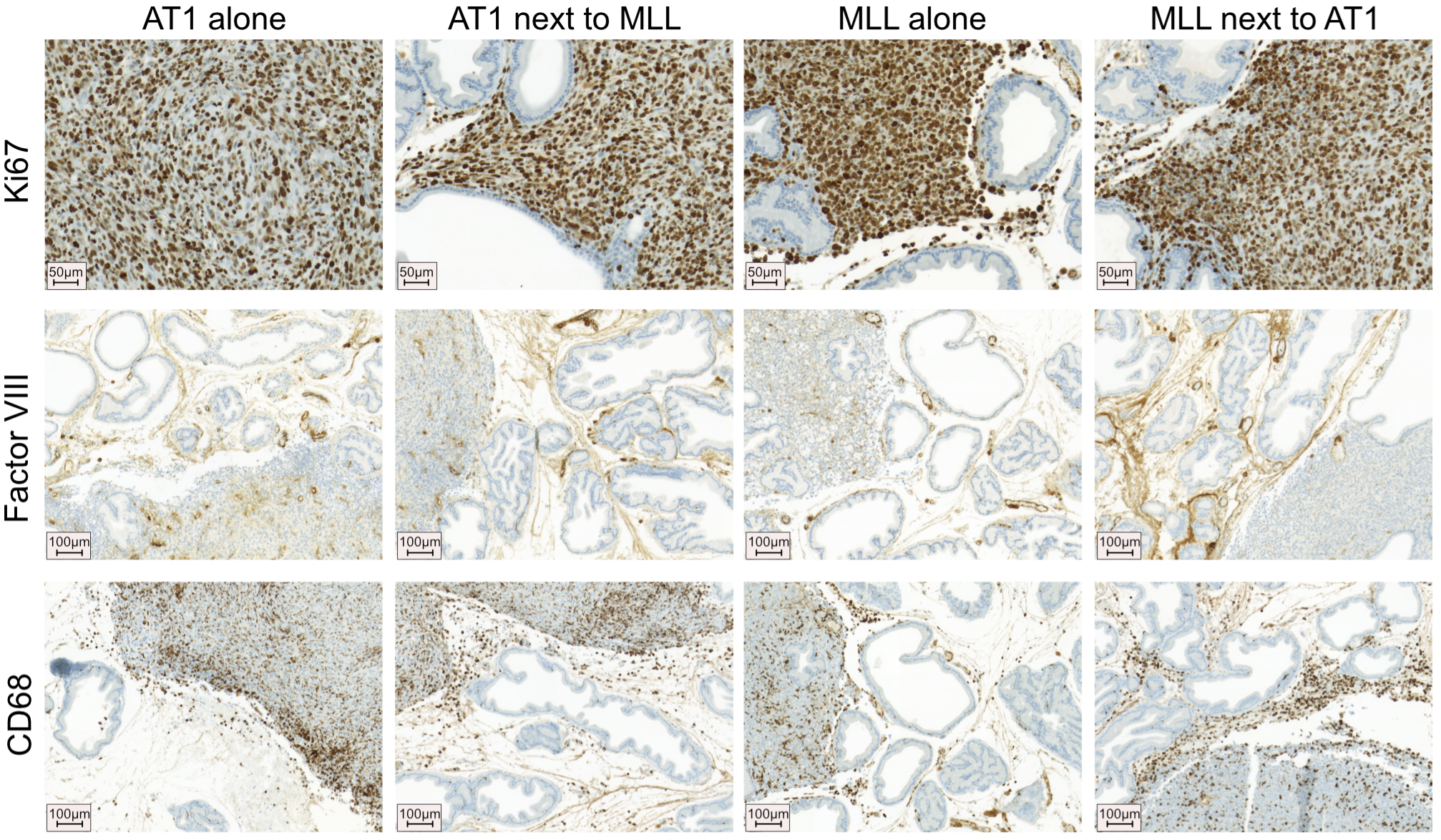

Supplement: Supplementary file 1 — Supplementary figure legends Figure S1. Immunostaining of intraprostatic rat tumours Figure S2. Morphology of lymph node metastases in rats Figure S3. Immunostaining of prostatectomy patient samples [file PATH-253-396-s001.zip › supp5604-sup-0001-FiguresS1-S3/path5604-sup-FigureS1.tiff]

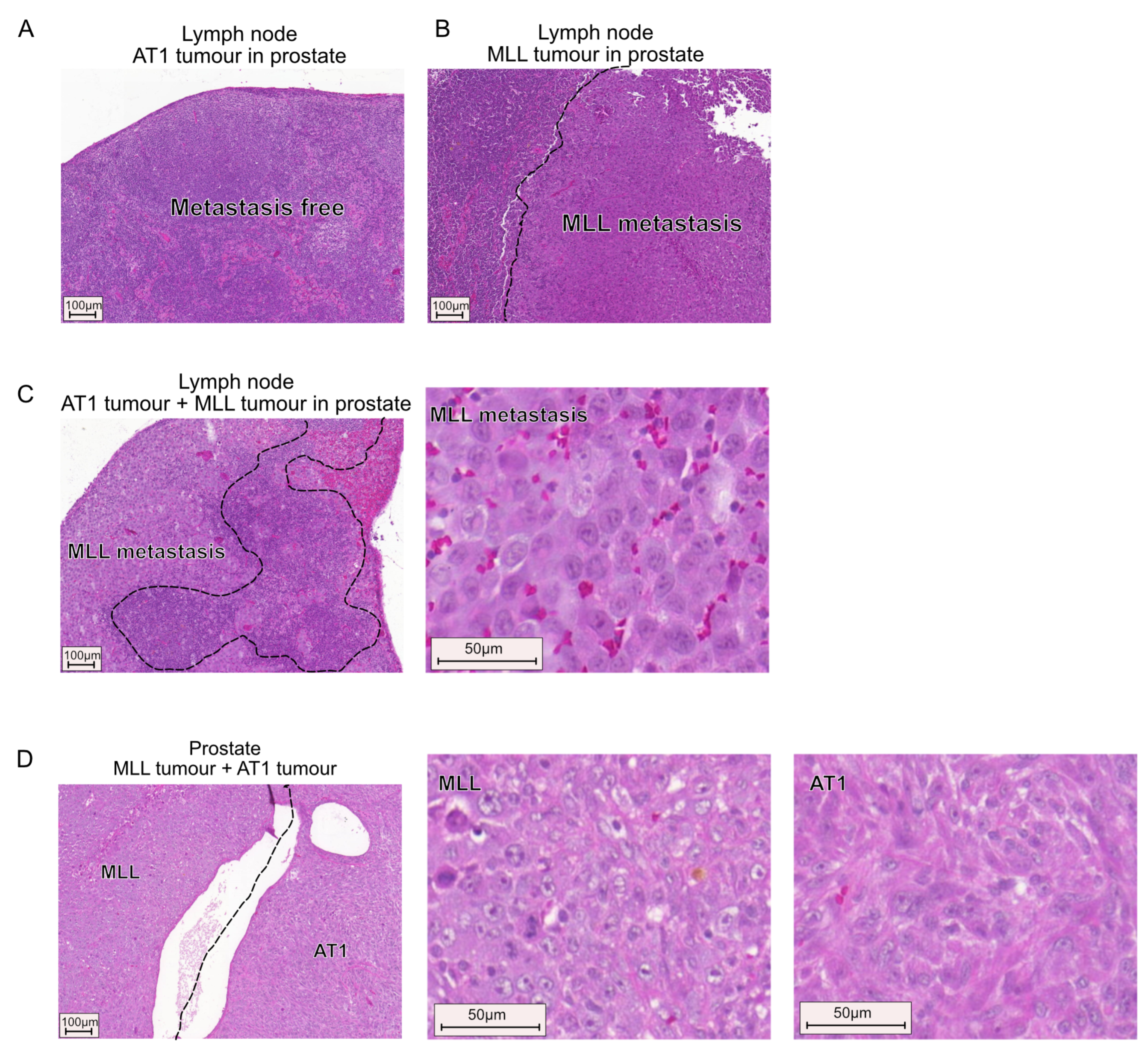

Supplement: Supplementary file 1 — Supplementary figure legends Figure S1. Immunostaining of intraprostatic rat tumours Figure S2. Morphology of lymph node metastases in rats Figure S3. Immunostaining of prostatectomy patient samples [file PATH-253-396-s001.zip › supp5604-sup-0001-FiguresS1-S3/path5604-sup-FigureS2.tiff]

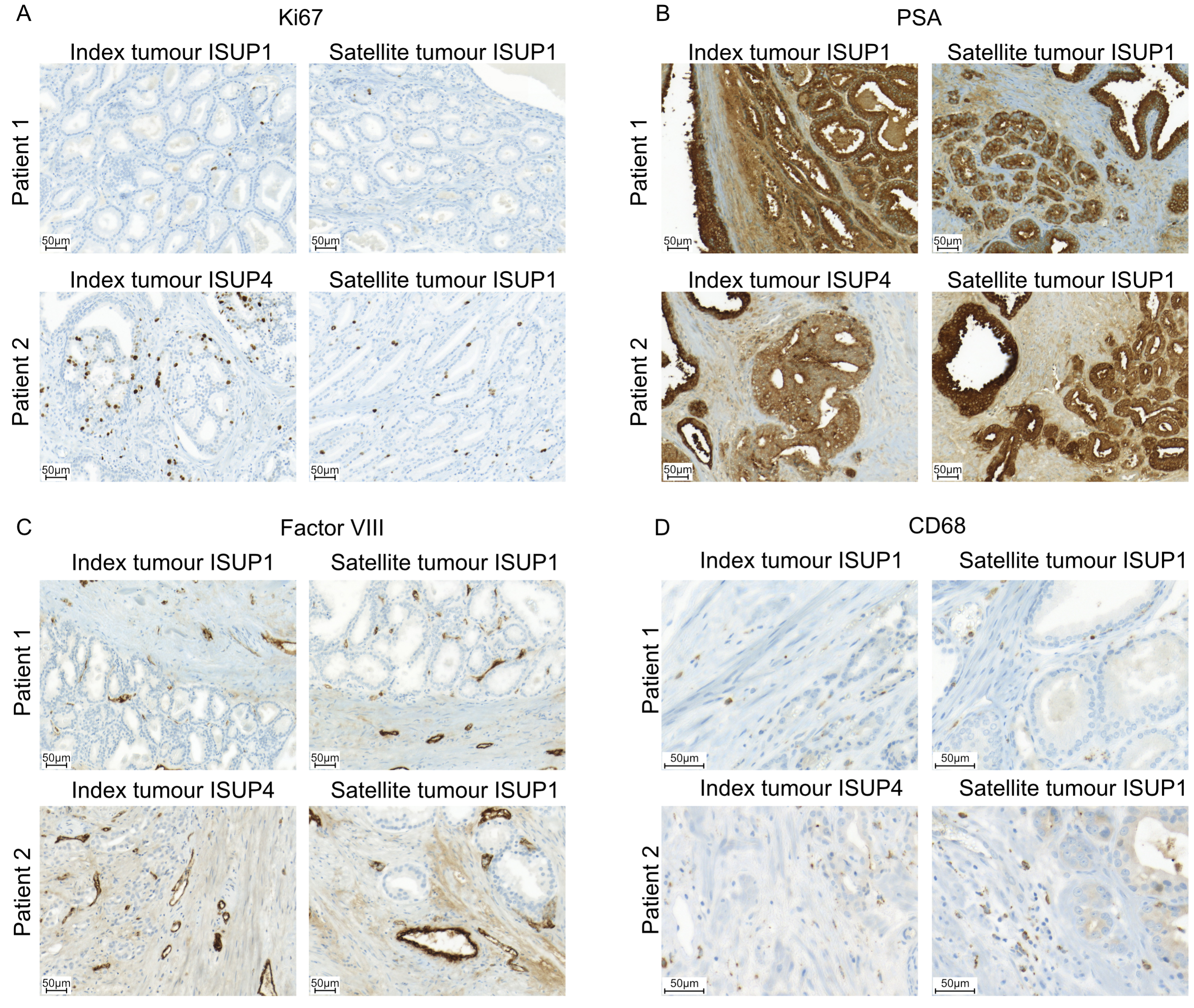

Supplement: Supplementary file 1 — Supplementary figure legends Figure S1. Immunostaining of intraprostatic rat tumours Figure S2. Morphology of lymph node metastases in rats Figure S3. Immunostaining of prostatectomy patient samples [file PATH-253-396-s001.zip › supp5604-sup-0001-FiguresS1-S3/path5604-sup-FigureS3.tiff]
